# *EjRAV1*/*2* Delay Flowering Through Transcriptional Repression of *EjFTs* and *EjSOC1s* in Loquat

**DOI:** 10.3389/fpls.2021.816086

**Published:** 2021-12-31

**Authors:** Ze Peng, Man Wang, Ling Zhang, Yuanyuan Jiang, Chongbin Zhao, Muhammad Qasim Shahid, Yunlu Bai, Jingjing Hao, Jiangrong Peng, Yongshun Gao, Wenbing Su, Xianghui Yang

**Affiliations:** ^1^State Key Laboratory for Conservation and Utilization of Subtropical Agro-bioresources, Key Laboratory of Innovation and Utilization of Horticultural Crop Resources in South China (Ministry of Agriculture and Rural Affairs), South China Agricultural University, Guangzhou, China; ^2^Fruit Research Institute, Fujian Academy of Agricultural Sciences, Fuzhou, China; ^3^Beijing Academy of Forestry and Pomology Sciences, Beijing Academy of Agriculture and Forestry Sciences, Beijing, China; ^4^College of Agriculture, South China Agricultural University, Guangzhou, China; ^5^Lushan Botanical Garden Jiangxi Province and Chinese Academy of Sciences, Lushan, China

**Keywords:** flowering time, juvenility, annual flowering, FT, branching, loquat, RAV

## Abstract

Most species in Rosaceae usually need to undergo several years of juvenile phase before the initiation of flowering. After 4–6 years’ juvenile phase, cultivated loquat (*Eriobotrya japonica*), a species in Rosaceae, enters the reproductive phase, blooms in the autumn and sets fruits during the winter. However, the mechanisms of the transition from a seedling to an adult tree remain obscure in loquat. The regulation networks controlling seasonal flowering are also largely unknown. Here, we report two *RELATED TO ABI3 AND VP1* (*RAV*) homologs controlling juvenility and seasonal flowering in loquat. The expressions of *EjRAV1/2* were relatively high during the juvenile or vegetative phase and low at the adult or reproductive phase. Overexpression of the two *EjRAV*s in *Arabidopsis* prolonged (about threefold) the juvenile period by repressing the expressions of flowering activator genes. Additionally, the transformed plants produced more lateral branches than the wild type plants. Molecular assays revealed that the nucleus localized EjRAVs could bind to the CAACA motif of the promoters of flower signal integrators, *EjFT1/2*, to repress their expression levels. These findings suggest that *EjRAVs* play critical roles in maintaining juvenility and repressing flower initiation in the early life cycle of loquat as well as in regulating seasonal flowering. Results from this study not only shed light on the control and maintenance of the juvenile phase, but also provided potential targets for manipulation of flowering time and accelerated breeding in loquat.

## Introduction

Flowering is a crucial process required for reproductive success, integrating external and internal signals (Song et al., 2013). This process is energy-consuming and therefore plants need to undergo diverse juvenile periods to accumulate sufficient reserves before flowering (Andrés and Coupland, 2012). For annual plants, such as *Arabidopsis*, the whole life cycle is limited to several months with a short juvenile phase followed by flowering and fruit setting in the adult phase, and the life cycle ends as fruits become mature (Somerville and Koornneef, 2002). In contrast, the long-lived perennial trees, such as poplar and eucalyptus, have a long juvenile phase that lasts for several years (Missiaggia et al., 2005; Hsu et al., 2006). Although perennial plants bloom annually or seasonally after the initiation of the first flowering, the long juvenile period is time-consuming and needs to be shortened to accelerate breeding and other research programs for economically important perennial plants (Watson et al., 2018).

As a key developmental event of a plant’s life cycle, the juvenile-to-adult transition of both annual *Arabidopsis* and perennial trees is regulated by gradually decreasing the *miR156/157* expressions as well as increasing the expressions of its target genes encoding a set of plant specific SPL (SQUAMOSA PROMOTER BINDING PROTEIN-LIKE) proteins (Xu et al., 2016; Jia et al., 2017). Commonly, the miR156 levels would decline as the plant age increases, which elevates the expression levels of SPL transcription factors to promote flowering by inducing the expressions of floral integrator genes including *FLOWERING LOCUS T* (*FT*), *SUPPRESSOR OF OVEREXPRESSION OF CO1* (*SOC1*), *APETALA1* (*AP1*), and *LEAFY* (*LFY*) (Wang, 2014; Xu et al., 2018).

The Rosaceae family possesses approximately 90 genera and 3,000 species with several fruit trees like apple, pear, *Prunus*, and strawberry producing delicious and nutritious fruits; ornamental plants such as rose, *Prunus mume* and oriental cherry as well as medicinal plants like loquat (also a fruit tree), *Potentilla chinensis* and hawthorn (Potter et al., 2007; Xiang et al., 2016). The growth habits vary greatly in this family, from herbaceous rosette plants to deciduous or evergreen trees. For example, the herbaceous strawberry could flower in its first season, while woody fruit trees usually need several years to end the juvenile period (Kurokura et al., 2013). FT, a major component of florigen, and its antagonistic homolog TERMINAL FLOWER 1 (TFL1) are vital regulators of plant flowering (Daniel, 2015), and their homologs in Rosaceae have proven to initiate the reproductive phase and regulate seasonal flowering (Kotoda et al., 2010; Kurokura et al., 2013). Overexpression of FT orthologs derived from fruit tree species in Arabidopsis induced its early flowering (Tränkner et al., 2010; Yarur et al., 2016). Currently available studies in fruit trees suggested the conserved role of FT in promoting the floral transition, and additional functions were implied in these studies based on the observations of pleiotropic transgenic phenotypes (Goeckeritz and Hollender, 2021). In some other fruit species such as kiwifruit, the FT orthologs were reported to play a role in maturity regulation and vegetative phenology in addition to flowering (Voogd et al., 2017). As another key flowering integrator, SOC1 was frequently reported to regulate the endodormancy break (Gómez-Soto et al., 2021). In sweet cherry, the *PavSOC1* gene was reported to interact with *dormancy-associated MADS-box* (*DAM*) genes to co-regulate flower development (Wang J. et al., 2020). In peach (*Prunus persica*), *PpSOC1* has been used as a marker for the timing of endodormancy break (Halász et al., 2021). Meanwhile, efforts have been made to use these genes as targets to shorten the juvenile period and accelerate breeding (Yamagishi et al., 2016; McGarry et al., 2017; Li et al., 2019). Nevertheless, the gene network regulating the initiation of flowering in Rosaceae is still rarely reported, resulting in limited novel gene targets.

Loquat (*Eriobotrya japonica*) is a subtropical evergreen fruit tree native to China (Su et al., 2021). The seedlings need to undergo 4–6 years of juvenile phase before the first flowering (Zheng et al., 1991). Its deciduous relatives, such as apple and pear, induce inflorescence in early summer, develop flower meristems in autumn, enter endodormancy in late autumn, and finally bloom in the next spring (Kurokura et al., 2013). By contrast, the cultivated loquat induces inflorescence and develops flower in the same year (Jiang et al., 2019), but the process from flowering to fruit setting occurs quickly in the winter season and fruits ripen in spring. The life history and annual growth cycle of cultivated loquat were illustrated in Supplementary Figure 1. As for loquat, previous works from our group and other researchers have identified some activators including *EjAP1* (Liu et al., 2013), *EdFT* and *EdFD* (Zhang et al., 2016), *EjLFY* (Liu et al., 2017), *EjSOC1* (Jiang et al., 2019), *EdCO* and *EdGI* (Zhang et al., 2019) and *EjSPL3*/*4/5/9* (Jiang et al., 2020a), as well as repressors like *EjTFL1* (Jiang et al., 2020b) and *EjFRI* (Chen W. et al., 2020). The functions of these genes were conserved between Arabidopsis and loquat. However, whether these genes or other genes in loquat have any relation with the control or maintenance of juvenility is still unknown. Meanwhile, the pathways of these genes to induce flowering remain to be elucidated.

*RELATED TO ABI3 AND VP1* (*RAV*) family genes, including *TEMPRANILLO* (*TEM*), play vital roles in linking photoperiod-, gibberellin- as well as other pathways to control flowering and juvenility in *Arabidopsis* (Castillejo and Pelaz, 2008; Osnato et al., 2012; Matias-Hernandez et al., 2014). However, it is unknown whether the homologs of *RAV* modulate flower initiation in long-lived perennial trees. To test this idea, here we cloned two *RAV* homologs (*EjRAV1* and *EjRAV2*) from *E. japonica*. *EjRAV1* and *EjRAV2* turned out to be good biomarkers that maintain juvenility and vegetative stages in loquat with abundant transcripts at these stages and rarely expressed at reproductive stages. In this study, we examined the roles of *EjRAV1* and *EjRAV2* in the transition from juvenile phase to reproductive phase in loquat, and investigated their functions in seasonal flowering in trees during fruit production. Our work revealed that EjRAV proteins could bind to the CAACA motifs in promoter regions of *FT* homologs to repress flower signal integration in loquat.

## Materials and Methods

### Plant Materials and Growth Conditions

According to our previous observations, the cultivated loquat trees start flower primordial differentiation and inflorescence formation from late June to July (Liu et al., 2013; Jiang et al., 2019). Hence, shoot apical meristem (SAM) observation as well as the collection of SAM and mature leaf samples of “Zaozhong-6,” a main cultivar in China, were performed from 16th May to 8th August in 2018 at the loquat germplasm resource preservation garden (South China Agricultural University, Guangzhou, China). The samples were collected at around 10:00–11:00 a.m. in every 7 days (except for 23rd May) during this time window. To decipher the correlations of gene expression levels between *EjRAV1/2* and other flowering regulating genes for the duration of juvenile phase, mature leaves from 1-year-old seedlings, 2-year-old seedlings, 2-year-old grafted trees and fruit-bearing adult trees (all trees with “Zaozhong-6” background) were collected on 11th July, 2018 (at around 10:00–11:00 a.m.) with three biological replicates from our germplasm resource preservation garden. For each replicate, three mature leaves were randomly collected from one plant and were immediately frozen in liquid nitrogen.

*Arabidopsis* and *Nicotiana benthamiana* were used for stable and transient genetic transformation, respectively. Columbia-0 (Col-0) ecotype *Arabidopsis* and *N. benthamiana* were preserved by our research group, and *tem2* (Col-0 background) mutant was purchased from the *Arabidopsis* Biological Resource Center^1^ with a stock number of CS909678. All these plants were planted in a long-day (16 h light/8 h dark) greenhouse at 22°C. The 4-week-old T3 *Arabidopsis* plants were collected for gene expression assays.

### *RAV* Gene Family Identification in Loquat Genome

The six RAV family protein sequences of *Arabidopsis* were downloaded from GenBank and used as queries to Blast search for homologs in “Jiefangzhong” loquat genome (Su et al., 2021) using TBtools software with an *E*-value cutoff at 1e^–10^, protein identity≥40 and bit score≥100 (Chen C. et al., 2020). Expression level heat maps of the six identified *EjRAV*s in 10 tissues were drawn based on FPKM values from transcriptome data (available at China National GeneBank database with accession number CNP0001531) using TBtools software (Chen C. et al., 2020). Multiple sequence alignment was performed using ClustalX^2^. The phylogenetic relationship of the *RAV* genes was calculated by MEGA5 (Tamura et al., 2011), with the bootstrap values at the branch nodes calculated from 100 replications. The following proteins were used for phylogenetic analysis: AtTEM/EDF1 (NP_173927.1), AtRAV2/EDF2/TEM2 (NP_173927.1), AtE DF3 (NP_189201.1), AtRAV1/EDF4 (NP_172784.1), AtRA V3 (CAA0284621.1), AtRAVL3 (NP_175524.1); CaRAV (AAQ05799.1), CsRAV1 (AEZ02303.1), DkERF32 (QFU8520 5.1), DkERF34 (QFU85207.1), EjRAV1 (QBQ58100.1), EjRAV2 (QBQ58101.1), FaRAV1 (AZL19498.1), FaRAV3 (AZL19 500.1), FaRAV5 (AZL19502.1), FaRAV6 (AZL19503.1), GmRAV (NP_001237600.1), MdRAV1 (MD13G1046100), MdRAV2 (AU Z96416.1), MtRAV1 (XP_003591822.1), MtRAV2 (AES97413.2), MtRAV3 (AES63108.1), NtRAV (NP_001311676.1), OsRAVL1 (ADJ57333.1), OsRAV6 (XP_015624169.1), PtRAV1 (XP_00 2315958.2), PtRAV2 (XP_002311438.2), PtRAV3 (XP_00230 4025.1), SlRAV2 (ABY57635.1). AtDRN-LIKE (NP_173864.1) was selected as an out-group.

### RNA Extraction and cDNA Preparation

Total RNA of loquat leaves and *Arabidopsis* plants were extracted with the EASYspin Plus plant RNA extraction kit (Aidlab, China) under the manufacturers’ instructions. Then, the PrimeScript™ RT reagent Kit (TaKaRa, Japan) was used to prepare the first-strand cDNA of all the samples.

### Gene Transformation in *Arabidopsis*

*EjRAV1* and *EjRAV2* sequences were amplified and cloned into the pGreen-35S vector to construct the pGreen-*35S:EjRAV1* and pGreen-*35S:EjRAV2* vectors with the primer pairs listed in Supplementary Table 1 using the PrimeSTAR^®^ HS DNA Polymerase (TaKaRa, Japan). The PCR amplicons and linearized vector (digested by *Hin*dIII and *Eco*RI; New England Biolabs, United States) were fused with a Hieff Clone^®^ Plus One Step Cloning Kit (Yeasen Biotech, China). *35S:EjRAV1* and *35S:EjRAV2* vectors were first transformed into the *Agrobacterium tumefaciens* GV3101:psoup cells, then floral-dip method was used to transfer overexpression vectors into *Arabidopsis* as previously described (Henriques et al., 2006).

### Quantitative Real-Time PCR Assays

Gene expression levels were analyzed using quantitative real-time PCR as previously done (Su et al., 2019). The specific quantitative primers were designed using the Primer Premier 6.0 software^3^. *EjRPL18* (MH196507) and *AtUBQ10* (AL161503) were used as the reference genes for loquat and *Arabidopsis* gene expression assays. Detailed primer sequence information for all the detected genes were listed in Supplementary Table 1. All the biological samples were detected in triplicates using iTaq™ universal SYBR Green Supermix (Bio-Rad, United States) in the LightCycler480 system (Roche, Sweden).

### Transient Expression Assays in *Nicotiana benthamiana*

The coding regions without stop codon of *EjRAV1* and *EjRAV2* were fused into pGreen-35S-GFP (green fluorescent protein) with primer pairs listed in Supplementary Table 1. The vectors were then transformed into *Agrobacterium tumefaciens* GV3101:psoup, GV3101:psoup cells at OD_600_ = 1.0 were centrifuged at 4,500 g for 5 min, and the pellets were suspended with MMA solution (MES, MgCl_2_ and Acetosyringone) to OD_600_ = 0.2 (Hellens et al., 2005). The dilute *Agrobacterium* cells were then incubated in dark under room temperature for 1 h and finally used to inject into *N. benthamiana* leaves. The tobacco leaves were incubated in long day greenhouse for 2 days, images of EjRAV1-GFP, EjRAV1-GFP and 35S-GFP were captured with the Observer. D1 fluorescence microscope system (Carl Zeiss, Germany).

The pGreen-0800:*EjSOC1* vectors were previously prepared (Jiang et al., 2020a). *EjFT1* and *EjFT2* promoter sequences were fused into pGreen-0800 vector with primer pairs listed in Supplementary Table 1, and the protocol for pGreen-0800 reporter vector construction was similar to that used in pGreen-*35S:EjRAV1* vector preparation. Then, PLACE website^4^ was used to check the binding sites for RAV proteins on each promoter. The positions of predicted RAV binding sites on these promoters were highlighted in Supplementary Figure 2 and listed in the Supplementary Table 2.

The coding regions of *EjRAV1* and *EjRAV2* were fused into the pSAK277 vector to construct the effector vector (Hellens et al., 2005) with primer pairs listed in Supplementary Table 1. The effector and reporter vectors were then transformed into *Agrobacterium tumefaciens* GV3101:psoup cells. Then, the mixed *Agrobacterium* solution with effector and reporter vectors was injected into *N. benthamiana* leaves as formerly performed (Hellens et al., 2005). The activities of firefly and renilla dual-luciferase were detected using a Dual-Luciferase Reporter Assay kit (Promega, America) under the instruction of the manufacturers 2–3 days after leaf injection.

### Electrophoretic Mobility Shift Assay

The coding sequences of *EjRAV1* and *EjRAV2* were cloned into pET28a using primers listed in Supplementary Table 1. The 6 × His-EjRAV recombinant proteins and 6 × His control were expressed in *Escherichia coli* BL21 (DE3) cells and induced at OD_600_ = 0.6∼0.8 by 1 mM isopropyl-beta-D-thiogalactoside (IPTG), and oscillated for 4 h at 220 rpm (revolutions per minute) after addition of IPTG. The raw induced proteins were then purified with Amylose Magnetic Beads (NEB, United States). Electrophoretic Mobility Shift Assay (EMSA) assays were performed as previously described (Yu et al., 2020). An EMSA Probe Biotin Labeling Kit (Beyotime, China) was then used to label biotin to the ends of oligonucleotide probes. Excessive unlabeled wild type or mutant probes were used for competition experiments. The probe sequences were listed in Supplementary Table 1. The EMSA assay was performed with an EMSA/Gel Shift kit and detected with a chemiluminescent EMSA kit (Beyotime, China). Ultimately, photos of all the gel shift experiments were taken with a ChimeScope series 3300 mini system (Clinxscience, China).

### Yeast One-Hybrid Assays

Yeast one-hybrid (Y1H) assays were performed according to the Matchmaker Gold Y1H system user manual (Clontech, America) to detect the binding capacities of *EjRAV1/2* to the *EjFT* promoters. Both *EjFT1* and *EjFT2* promoters contain five putative RAV binding elements (CAACA motif or CACCTG motif, see in Supplementary Figure 2 and Supplementary Table 2). According to Supplementary Figure 2, four promoter segments containing all the binding motifs were designed for yeast binding assays, and the segment regions were separately inserted into the pAbAi reporter vector to obtain baits. Meanwhile, the CDS of *EjRAV1/2* was fused to the pGADT7 vector. Then, the recombinant pGADT7-EjRAV1 or pGADT7-EjRAV2 was transformed into the Y1HGold yeast strain with linearized reporter plasmid pAbAi-proFT1 or pAbAi-proFT2 to further determine the protein–DNA interactions. The primers used in these assays were listed in Supplementary Table 1.

## Results

### Identification of *RAV* Family Genes

**FIGURE 1:**
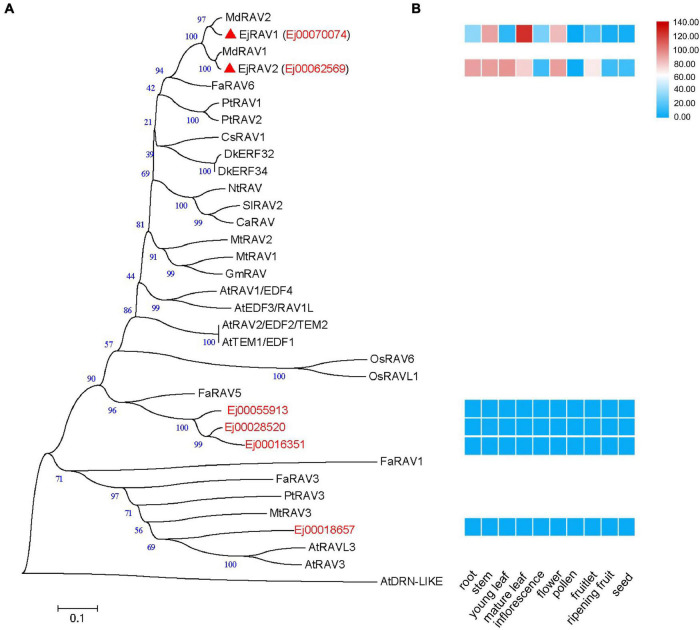
A phylogenetic tree and expression heat map of *RAV* family genes in loquat. **(A)** A phylogenetic tree containing *RAV* homologs identified in loquat genome and other species. AtDRN-LIKE was selected as an outer-group. The red triangles indicate candidate *RAVs* that play role in the transition of reproductive phase in loquat. At, *Arabidopsis thaliana*; Ca, *Capsicum annuum*; Cs, *Castanea sativa*; Dk, *Diospyros kaki*; Ej, *Eriobotrya japonica*; Fa, *Fragaria ananassa*; Gm, *Glycine max*; Md, *Malus domestic*a; Mt, *Medicago truncatula*; Nt, *Nicotiana tabacum*; Os, *Oryza sativa*; Pt, *Populus trichocarpa*; Sl, *Solanum lycopersicum*. **(B)** Tissue expression patterns of *EjRAV* family genes based on FPKM values from 10 tissues.

A total of six *RAV* family genes were identified in the loquat genome (Figure 1A). These genes were clustered into three clades, among which Ej00070074 and Ej00062569 were clustered with four *Arabidopsis RAV*/*TEM* genes (*RAV1*, *RAV1-LIKE*, *RAV2* and *RAV2-LIKE*), Ej00055913, Ej00028520 and Ej00016351 grouped with *FaRAV5*, while Ej00018657 clustered into the *RAV3* clade. These genes showed different expression patterns. Ej00070074 and Ej00062569 (formerly named as *EjRAV1* and *EjRAV2*) were found to be highly expressed in most of the vegetative tissues while the expression levels of the other four genes were scarcely detected (Figure 1B). The highly expressed *EjRAV1* and *EjRAV2* were speculated to possibly play a role in repressing flowering or repressing flower bud differentiation in loquat.

### *EjRAV1/2* Abundance Negatively Associated With the Annual Flowering Pattern

**FIGURE 2:**
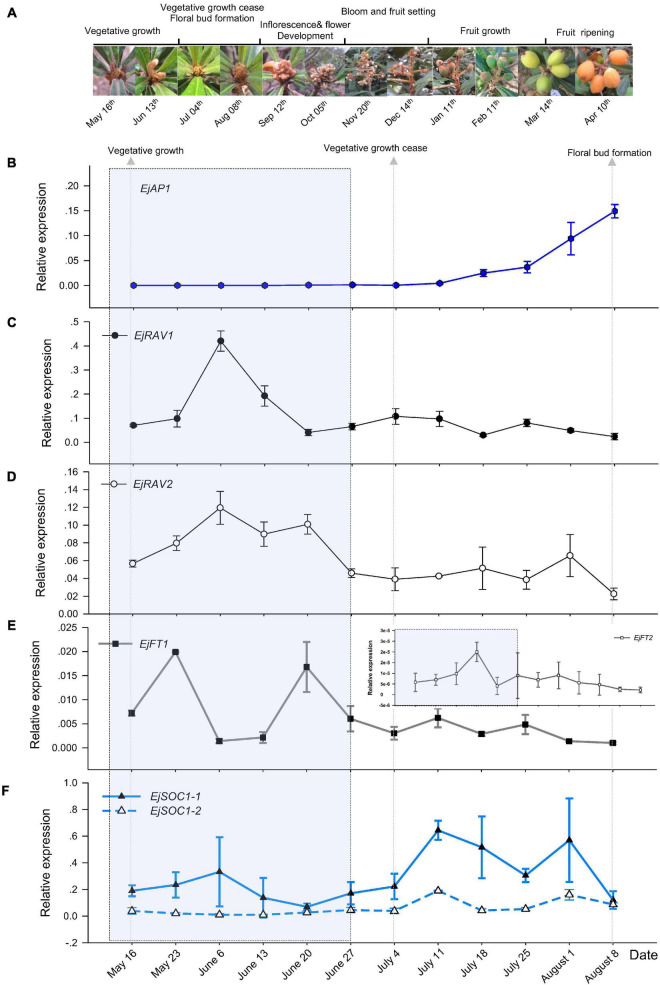
Floral initiation and expressions of flowering regulating gene homologs in “Zaozhong-6” loquat. **(A)** Developmental changes in shoot apical meristem (SAM) of “Zaozhong-6” loquat from May 2018 to April 2019. **(B–F)** Expression patterns of *EjAP1* **(B)**, *EjRAV1* **(C)**, *EjRAV2* **(D)**, *EjFT1/2* **(E)**, and *EjSOC1-1/2* **(F)** during floral initiation and early inflorescence development. *EjAP1* was detected in SAM, while other genes were detected in mature leaves. The error bars represent standard deviation for three biological replicates. The light gray colored window indicates vegetative phase.

To understand the potential roles of *EjRAV1* and *EjRAV2*, we first analyzed their expression patterns in fruit producing trees of Zaozhong-6 during inflorescence development. We started observations in May because fruits ripened in April and the SAM was still at vegetative growth stage. At this moment, the expression of *EjAP1*, a marker gene for floral initiation, was almost undetectable (Figures 2A,B). The SAM of trees stopped growing in July as the vegetative growth ceased, and the floral initiation and inflorescence development started. Ultimately, the SAM was transformed into inflorescence from shoot as the transcription levels of *EjAP1* started to elevate dramatically from 18th July (Figures 2A,B).

Abundant *EjRAV1* and *EjRAV2* transcripts were detected at the vegetative growth stage. When the vegetative bud growth ceased, their expression levels were dramatically decreased and maintained at extremely low levels till the floral initiation started (Figures 2C,D). In contrast, the expressions levels of *EjFT1* and *EjFT2* increased as *EjRAV1* and *EjRAV2* were down-regulated (Figure 2E). Interestingly, the expression levels of both *EjSOC1-1* and *EjSOC1-2* increased after the up-regulation of *EjFT1* and *EjFT2*, and a remarkable increase in the expression levels of *EjAP1* was also detected (Figure 2F). The gene expression assays suggested that *EjRAV1* and *EjRAV2* may act as negative regulatory factors in the initiation of flowering in a fruit producing loquat tree.

### Abundant *EjRAV1/2* and Rare *EjFT* and *EjSOC1* Transcription Levels in Seedlings at Juvenile Phase

**FIGURE 3:**
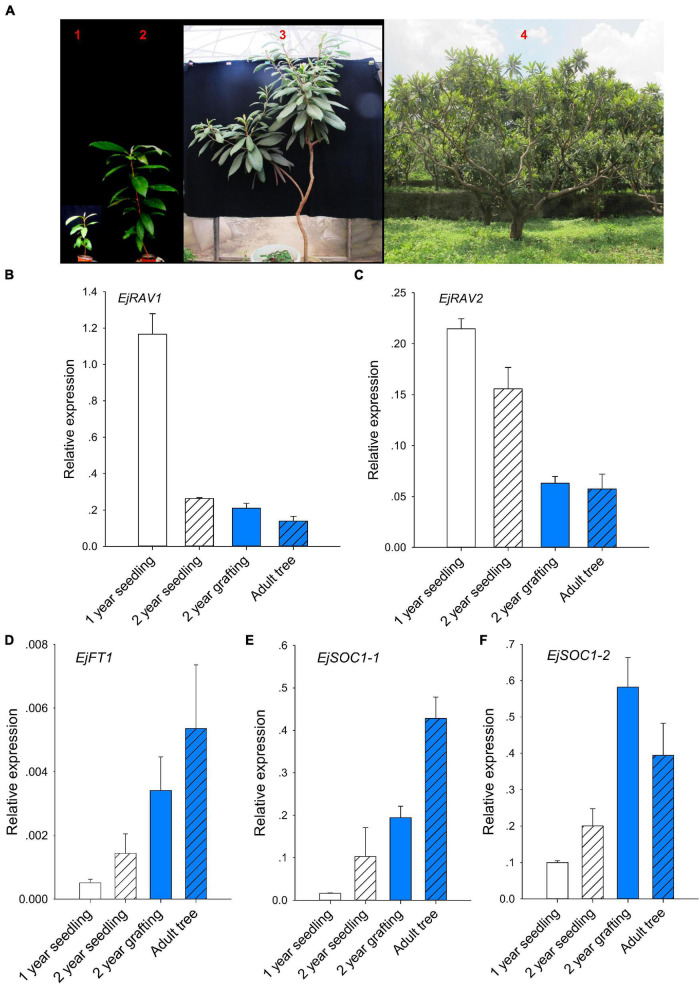
Association of abundant *EjRAVs* expressions and rare flowering activator gene expressions with the juvenile phase. **(A)** “Zaozhong-6” plants at different developmental stages in July, 2018. The red numbers, 1–4, in *A* showed plants of a 1-year-old seedling, a 2-year-old seedling, a 2-year-old grafted tree and an adult fruit-bearing tree used for gene expression assays, respectively. (**B–F**) Expression patterns of *EjRAV1* **(B)**, *EjRAV2* **(C)**, *EjFT1* **(D)**, *EjSOC1-1* **(E)**, and *EjSOC1-2* **(F)** in mature leaves of these trees. The expression level of *EjFT2* was almost undetectable in all the trees and data were not shown. The error bars in **(B–F)** represent standard deviation for three biological replicates.

Prolonged juvenile phase is one of the most important ways that guarantee species survival. However, it reduces breeding efficiency, especially for perennial crops. To further confirm whether *EjRAV1* and *EjRAV2* play roles in the regulation of juvenile phase in loquat, we analyzed the expression patterns of *EjRAV1*/*2* and other important flowering regulating genes in 1-year-old seedlings, 2-year-old seedlings, 2-year-old grafted trees, and fruit-bearing trees (Figure 3A). The 1-year-old seedlings and 2-year-old seedlings were still at the juvenile phase. The SAMs of both 2-year-old grafted trees (the scions were cut from fruit-bearing trees) and fruit-bearing trees were transformed into inflorescence in the autumn and set fruits later. The abundance of *EjRAV1* and *EjRAV2* transcripts were gradually reduced as the tree achieved the ability to form floral bud step by step (Figures 3B,C). By contrast, the expression levels of *EjFT1*, *EjSOC1-1* and *EjSOC1-2* increased continuously over this period (Figures 3D–F). Overall, the gene expression analyses suggested that *EjRAV1* and *EjRAV2* may act as important regulators of juvenility phase by repressing the flowering activator gene expressions.

### Overexpression of *EjRAV1*/*2* Prolonged the Juvenile Phase in *Arabidopsis*

**FIGURE 4:**
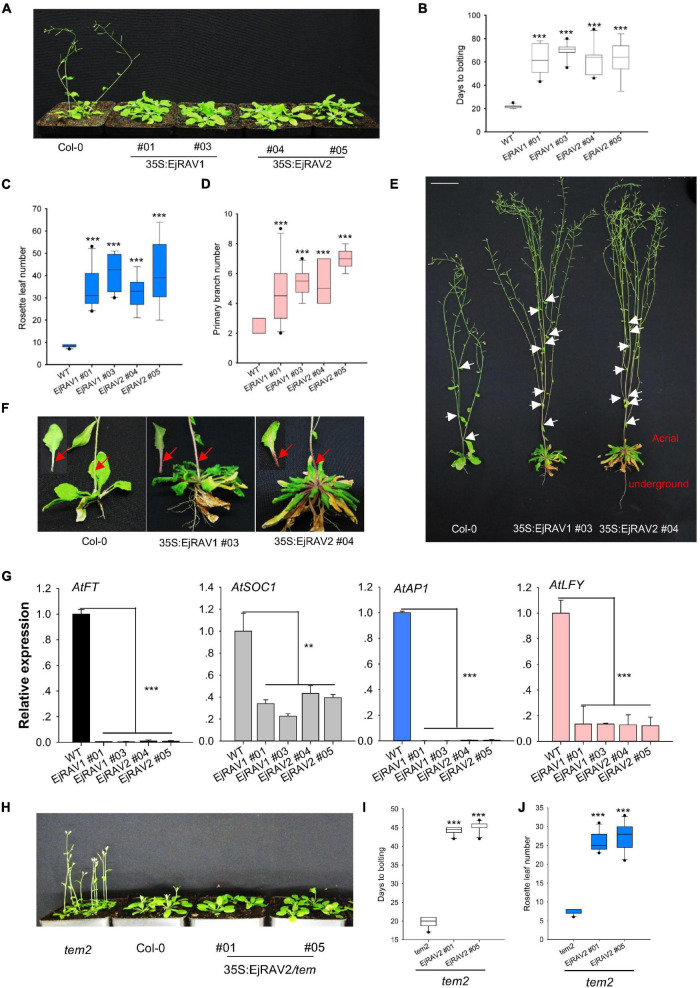
Overexpression of *EjRAV1* and *EjRAV2* delay flowering and promote stem branching in *Arabidopsis thaliana*. **(A)** *EjRAV1-*OE and *EjRAV2-*OE delay flowering in Col-0 ecotype *Arabidopsis*. **(B)** The juvenile length (days to bolting) of all the overexpressed lines were strikingly prolonged. **(C)** The numbers of rosette leaves were greatly increased in the *EjRAV1/2*-OE lines. **(D)** *EjRAV1-*OE and *EjRAV2-*OE promoted lateral branching. **(E)** The phenotypes of Col-0 and *EjRAV1/2*-OE transgenic *Arabidopsis* plants. The ectopic expression of *EjRAV1/2* was not only involved in the regulation of plant architecture (see leaf floral and axis branch numbers) but also associated with strong root system. Arrows indicate the growing point of the primary branch. **(F)** *EjRAV1/2*-OE activated anthocyanin accumulation in petiole and stem. Arrows represent abundant anthocyanin enriched in the basal parts of petiole and stem. **(G)** *EjRAV1-*OE and *EjRAV2-*OE repressed the expressions of flower inducer genes (*FT*, *SOC1*, *AP1*, and *LFY*) in *Arabidopsis*. **(H)** *EjRAV2*-OE rescues the phenotype of *tem2* mutant (Col-0 background) to Col-0 at 4 weeks post germination. The **(I)** juvenile lengths and **(J)** rosette leaf numbers of the *EjRAV2*-OE transgenic plants (on *tem2* background). The ** and *** indicate significant differences at *P* < 0.01 and < 0.001. The error bars in **(B–D,I,J)** represent standard deviation for 15 T_3_ plants. The error bars in **(G)** represent standard deviation for three replicates.

To confirm the above-mentioned speculation on the functions of *EjRAV1* and *EjRAV2* in the regulation of flowering time, we cloned each of them into a pGreen-35S plant overexpression vector for overexpression (OE) in *Arabidopsis*. Sequence alignment revealed that the encoded proteins of both genes are characterized by an AP2 domain and a B3 domain, as well as by a nuclear localization signal (NLS) and a B3 repression domain (Supplementary Figure 3). OE of both *EjRAV1* and *EjRAV2* in Columbia-0 (Col-0) background significantly delayed flowering (Figure 4A). The days to bolting after germination strikingly increased (3–4-fold) for all the OE transgenic plants than the wild type plants (Figure 4B), which indicated that *EjRAV1* and *EjRAV2* prolonged the juvenile phase in transgenic plants. At the same time, the number of rosette leaves of each OE transgenic line also greatly increased (Figure 4C). Unexpectedly, all the OE transgenic plants had a larger number of branches (about twofold) (Figures 4D,E) and stronger root systems than that of Col-0 (Figure 4E). It was also interesting that abundant anthocyanins were enriched in the basal parts of petiole and stem for all the OE transgenic plants (Figure 4F). Gene expression quantification confirmed that the expression levels of flowering activating genes, including *AtFT*, *AtSOC1*, *AtAP1*, and *AtLFY*, were significantly reduced in EjRAV1-OE and EjRAV2-OE transgenic plants compared to the wild type plants (Figure 4G). In addition, *EjRAV1* and *EjRAV2* were overexpressed in the *tem2* mutant. Only stable EjRAV2-OE lines were obtained on the *tem2* background. EjRAV2-OE/*tem2* rescued the phenotype of *tem2* to that of Col-0 wild type plant (Figure 4H). The days to bolting and numbers of rosette leaves of EjRAV2-OE/*tem2* transgenic plants were strikingly larger (> 2 fold) than that of the *tem2* mutant (Figures 4I,J). In summary, the OE experiments conducted here indicated that *EjRAV1* and *EjRAV2* repress the flowering and activate branching and anthocyanin accumulation in transgenic plants.

### *EjRAV1/2* Localized in Cell Nuclei and Bind to CAACA Motifs to Repress the Expressions of *EjFT* and *EjSOC1*

**FIGURE 5:**
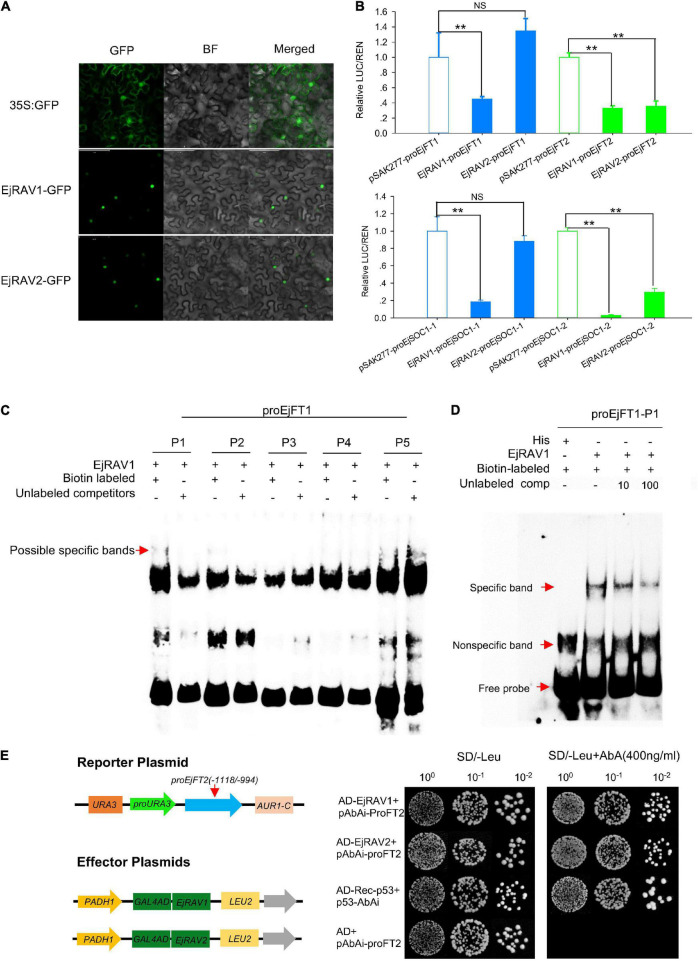
Protein localization, binding and regulation abilities of EjRAVs on FT and SOC1 homologs in loquat. **(A)** Subcellular localization of *EjRAV1/2* proteins expressed in tobacco epidermal cells. **(B)** *EjRAV1/2* regulation on *EjFT*s and *EjSOC1*s expressions in *N. benthamiana* leaves by using the dual-luciferase (LUC) system. Relative renilla (REN) luciferase activity was used as an internal control. Data represent means ± standard error from three replicates. **P < 0.01 and NS indicate significance and non-significance, respectively. **(C,D)** Binding ability of EjRAV1 to P1 of *EjFT1* promoter in EMSA assays. P1, P2, P3, P4, and P5 are presumptive RAV protein binding sites on promoter of *EjFT1* at (−1583 to −1579 bp), (−1059 to −1054 bp), (−1033 to −1029 bp), (−782 to −778 bp), and (−73 to −66 bp). Unlabeled probe could reduce binding ability of EjRAV1 to P1 in **(D)**. **(E)** Binding ability of EjRAV1 and EjRAV2 to segment3 (−1118 to −994 bp) of *EjFT2* promoter in Y1H assays.

In agreement with the conserved NLS) appeared at the 5’ terminals of the encoded amino acid sequences (Supplementary Figure 3), EjRAV1-GFP and EjRAV2-GFP were localized in the cell nuclei of tobacco leaf cells (Figure 5A). To understand whether these two proteins play a role in *EjFT* or *EjSOC1* expression modulation, dual-luciferase assays were performed. A total of 5, 5, 5, and 1 candidate RAV family protein binding sites were identified in the promoter regions of *EjFT1*, *EjFT2*, *EjSOC1-1* and *EjSOC1-2*, respectively (Supplementary Figure 2 and Supplementary Table 2). The luciferase assay data showed that EjRAV1 greatly reduced the expressions of *EjFT1/2* and *EjSOC1-1/2*, while EjRAV2 could significantly inhibit the expressions of *EjFT2* and *EjSOC1-2* (Figure 5B). Overall, these results suggested that the *EjRAV1/2* proteins localized in the cell nucleus play regulatory roles in loquat.

To further check the abilities of EjRAV1 and/or EjRAV2 to regulate the expression levels of these genes, EjRAV1 and EjRAV2 proteins were prepared with *Escherichia coli* cells. Specific bands were induced by IPTG with protein size between 40 and 55 KDa and purified with Amylose Magnetic Beads. Protein-DNA binding was then assayed for EjRAV1-*proEjFT1/2* and EjRAV2-*proEjFT1/2* (Supplementary Figure 4), EjRAV1-*proEjSOC1-1/2* and EjRAV2-*proEjSOC1-1/2* (Supplementary Figure 5) combinations. The EMSA assays indicated that EjRAV1, but not EjRAV2, can bind directly to the CAACA motif at P1 (−1583 to −1579 bp) of *proEjFT1*, but not *proEjFT2, in vitro* (Figures 5C,D and Supplementary Figure 4). Yeast one-hybrid assay was performed to identify other binding sites on *proEjFT2*, which showed that EjRAV1 and EjRAV2 can directly bind *in vivo* to segment3 (−1118 to −994 bp) that contains two CAACA motifs (Figure 5E).

## Discussion

### Loquat, a Distinct Evergreen Fruit Tree, Blooms in Autumn

The decision to flower in plants during both the early life cycle and annual growth season is a complicated process that ultimately affects reproductive ability, breeding efficiency as well as other research progresses on the species (Somerville and Koornneef, 2002). In addition, flowering time determines the season for fruit, cut-flower and other horticultural products supplies (Wilkie et al., 2008). Loquat is one of the minor fruit trees whose fruits ripen from spring to early summer to meet the market demand of fresh fruits in spring, which benefits the growers greatly (Badenes et al., 2009). Commonly, SAM of perennial plants such as poplar (Tylewicz et al., 2018) and deciduous fruit trees (Kurokura et al., 2013) would cease growth and enter dormancy in fall to enable survival in the cold-drought stressful winter. The time of dormancy release is closely related to the flowering time of deciduous fruit trees including apple, pear, peach, and apricot (Falavigna et al., 2019). Here, we found that loquat initiated floral bud formation (Figure 2A) in a similar season compared with that of apple and pear in summer (Wilkie et al., 2008); however, the loquat SAM developed into an indeterminate panicle and bloomed immediately after floral initiation (Figure 2A) without the growth cease of reproductive buds or entering dormancy in fall. These results were in accordance with previous observations on the morphological transition of SAM, which also revealed immediate floral initiation in loquat (Liu et al., 2013; Jiang et al., 2019). Collectively, the results from the present and previous studies revealed that the constant development of inflorescence and flower bud without entering dormancy leads to the unique flowering habit (in autumn) of cultivated loquat. Future in-depth studies are required to elucidate how cultivated loquat has evolved this distinct flowering ability.

### *RAV* Homologs Maintain Juvenility and Repress Floral Induction in Loquat

The peculiar flowering habit of loquat ensures the availability of its fresh fruits in spring, a season short of fresh fruits in the market. Lots of endeavors aiming to identify pathways modulating flower induction in loquat have been carried out in the last decade (Esumi et al., 2005; Yang et al., 2007; Fernández et al., 2010; Liu et al., 2013; Zhang et al., 2016; Reig et al., 2017; Jiang et al., 2019, 2020a,b; Chen W. et al., 2020; Xia et al., 2020). *FT* and *SOC1* are floral integrators that link upstream photoperiod and other environmental or internal signals to promote flowering, while the downstream *AP1* controls the identity of floral meristem during flower bud transition (Blümel et al., 2015; Bouché et al., 2016). In loquat, using orthologs of these genes as biomarkers, the developmental stages of SAM during the annual growth cycle were classified as vegetative growth, vegetative growth cease and floral bud initiation (Figure 2), in accordance with our former histological observations (Jiang et al., 2019). In reference to these stages, negative correlations were detected between the expressions of *EjRAV1/2* and the flowering activators, *FT*, *SOC1*, and *AP1*. Notably, the elevation of expressions of flowering activators occurred after the down-regulation of *EjRAV1/2* (Figures 2B–F). These results implied that *EjRAV1/2* are repressors of flowering during the vegetative phase of an annual growth season.

The long juvenile phase would severely reduce breeding efficiency of a perennial crop. Therefore, identifying targets to promote flower initiation at early stage became utmost important (Watson et al., 2018). Although a few flowering regulating genes have been reported to shorten the juvenile period (Hsu et al., 2006; Kotoda et al., 2010; Li et al., 2019), few studies were focused on the identification of candidate genes associated with the promotion or release of juvenility (Missiaggia et al., 2005). In the current study, remarkable negative correlations were discovered between the expressions of *EjRAV1/2* and three flowering activator genes in loquat trees at different growth stages. The abundant accumulation of *EjRAV1/2* was related with the low expressions of *EjFT* and *EjSOC1*s at seedling stage, while high expression levels of *EjFT* and *EjSOC1*s and low levels of *EjRAV1/2* were detected in fruit bearing trees. Similarly in olive and antirrhinum, high *DeTEM* or *AmTEM* gene expressions were detected during the juvenile phase (Sgamma et al., 2015). To further validate the functions of *EjRAV1/2*, we generated overexpressed *EjRAV1* and *EjRAV2* transgenic plants of *Arabidopsis* with two different backgrounds (Col-0 ecotype and *tem2*), and the flowering time of all the transgenic plants were delayed while the juvenile period prolonged strikingly with a sharp decline of expression levels of flowering activator genes (Figure 4G). Our results were consistent with previous studies on the overexpression of *AtTEM1*/*AtTEM2* (Castillejo and Pelaz, 2008) and *DeTEM*/*AmTEM* (Sgamma et al., 2015). Here, the gene expression analyses and overexpression experiments revealed that *EjRAV1/2* are vital regulators of prolonged juvenility and repress floral induction in loquat.

### EjRAVs Bind to the CAACA Motif of Flowering Signal Integrators to Delay Flower Bud Induction

Binding to the CAACA or CACCTG motif is essential for *RAV* genes to link photoperiod and gibberellin pathways to modulate flowering in *Arabidopsis* (Kagaya et al., 1999; Castillejo and Pelaz, 2008; Osnato et al., 2012). The expression of *FT*, modulated by the balance between CO and TEM, is an integrated signal that triggers flowering (Castillejo and Pelaz, 2008). Here, we identified the binding and transcriptional regulation capacities of *EjRAV1/2* on *FT* and *SOC1* homologs. Our results confirmed that cell nuclei localized *EjRAV1/2* bind to the CAACA motif upstream of *EjFT1* and *EjFT2* promoters to repress their expressions. Although SOC1 is also a flowering pathway integrator and *EjRAV1/2* sharply reduced the expression of *EjSOC1*, no binding site was detected on *EjSOC1*’s promoter region by EMSA (Supplementary Figure 5). This might be due to that *EjRAV1/2* influence *EjSOC1*’s expression through another way instead of directly binding to the promoter region of EjSOC1, or due to other reasons, which requires future investigations. While AtRAVs and EjRAVs bind to the CAACA motifs upstream of *FT* promoter regions (Castillejo and Pelaz, 2008), OsRAVL1, a RAV homolog from rice, binds to the E-Box (CACCTG) in the promoters of Brassinosteroid (BR) signaling and the biosynthetic genes (Je et al., 2010).

### *EjRAVs* Promote Branching and Anthocyanin Accumulation

Plant architecture is one of the most important factors modulating grain/fruit production (Wang L. et al., 2020). Loquat is particularly robust in apical dominance and deficient of lateral branches, which remarkably limit the potential of fruit yield improvement (Badenes et al., 2009). Interestingly, the overexpression of *EjRAV1/2* remarkably promoted lateral branches in *Arabidopsis*, similar to the role of their close homologs *CsRAV1* (Moreno-Cortés et al., 2012) and *PtRAV1* (Moreno-Cortés et al., 2017) in the induction of sylleptic branches. Transgenic plants overexpressing *RAV1* lost shoot apical dominance in *Arabidopsis*. In rice, *OsRAVL1* (Je et al., 2010) and *OsRAV6* (Xianwei et al., 2015) maintain BR homeostasis to modulate leaf angle and seed size. Meanwhile, the *TEM* genes in *Arabidopsis* can link photoperiod and gibberellin signals to control flowering (Osnato et al., 2012) and jasmonic acid signaling in salt tolerance (Osnato et al., 2020). These studies suggest that *RAVs* may act to reprogram hormone homeostasis and signal transduction to further promote lateral branches. In *Arabidopsis*, changes on strigolactone signaling affected shoot branching and anthocyanin biosynthesis *via* binding to *BRANCHED1* (*BRC1*) and *PRODUCTION OF ANTHOCYANIN PIGMENT 1* (*PAP1*) promoters (Wang L. et al., 2020). Consistent with this speculation, research in strawberry revealed that *FaRAV1* can positively regulate anthocyanin accumulation in the fruit by activating *FaMYB10*, a homolog of *PAP1* (Zhang et al., 2020). Meanwhile, the vital branching regulators including *BRC1* (Niwa et al., 2013) and it homologs *TEOSINTE BRANCHED 1*, *CYCLOIDEA*, *PCF/TCP* transcription factors (Lucero et al., 2017) were involved in flowering time regulation *via* interacting with *FT* or direct regulation of *SOC1* transcription. Therefore, the *RAV* gene family play vital roles in many developmental processes.

**FIGURE 6:**
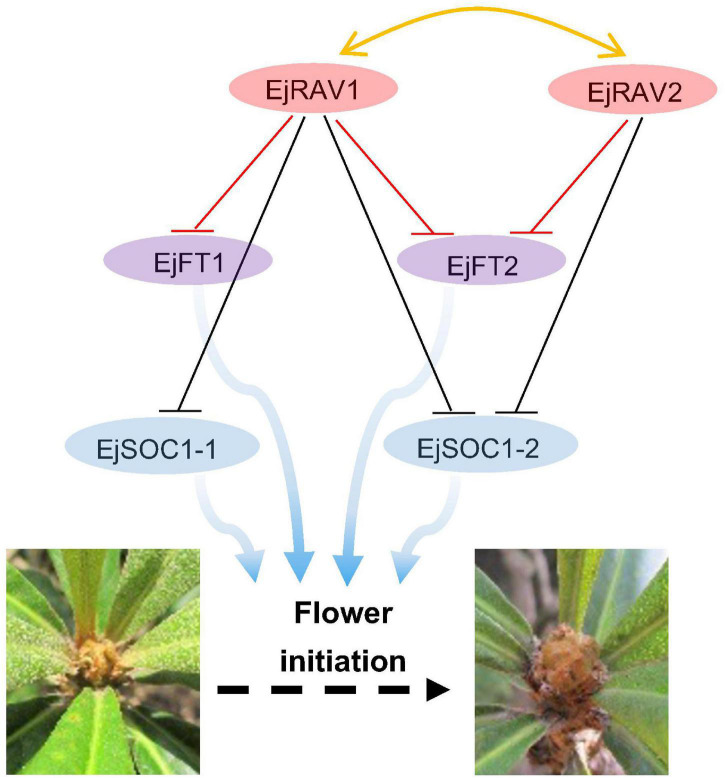
A proposed model for *EjRAV1/2* to regulate flowering time. The central network shows that *EjRAV1/2* repress *FT* and *SOC1* homologs expressions to delay flowering in an annual production season of loquat. The arrows represent promotion of FT and SOC1 homologs on flower initiation. The T arrows represent transcriptional repression of *EjRAV1/2* on *FT* and *SOC1* expressions.

Here, we identified two multifunctional *RAV* homologs in loquat with major roles in maintaining juvenility and delaying flowering in an annual growth cycle. Based on these results, we presented a potential model of pathways of *EjRAV1/2* to modulate floral bud formation during the tree life cycle and annual growth cycle based on molecular assays (Figure 6). In this putative model, *EjRAV1/2* bind directly to the CAACA motifs upstream of *EjFT1* and *EjFT2* promoters and repress their expressions to keep loquat trees in the juvenile phase and delay flowering in an annual growth cycle. In addition, *EjRAV1* and *EjRAV2* are important regulators of shoot branching and anthocyanin biosynthesis. The substantial evidence revealed that *EjRAV1/2* play vital roles in the initiation of flowering, and both could be potential targets for breeding programs to accelerate loquat breeding by shortening the juvenile phase and extend the fruit supply season.

## Conclusion

Two *RELATED TO ABI3 AND VP1 (RAV)* homologs were isolated in loquat. These two genes maintained high expressions during the juvenile or vegetative phase, which was negatively correlated with flowering promoting genes like *EjFT1*/*EjFT2* and *EjSOC1-1/EjSOC1-2*. Overexpression of *EjRAV1* and *EjRAV2* delayed flowering and repressed *FT*, *SOC1* and *AP1* expressions in *Arabidopsis*. Both EjRAV1 and EjRAV2 proteins are localized to the cell nuclei and could bind to the CAACA motifs and repress the expressions of *EjFTs* and *EjSOC1s*. These findings contribute to our understanding of the molecular mechanism of loquat phase change and flowering time regulation and provide gene targets to shorten the fruit tree juvenility and accelerate breeding.

## Data Availability Statement

The original contributions presented in the study are included in the article/Supplementary Material, further inquiries can be directed to the corresponding author/s.

## Author Contributions

XY, WS, and YG designed the research and obtained the funding. MW and WS performed the transgenic transformation and Y1H assays. MW, WS, and YJ performed the luciferases reporter assays. LZ and WS prepared the proteins and performed the EMSA assays. MW, ZP, WS, CZ, YB, JH, JP, and YG performed the other experiments. WS, MW, and ZP analyzed the data. ZP, WS, XY, and MS prepared the manuscript. All authors contributed to the article and approved the submitted version.

## Conflict of Interest

The authors declare that the research was conducted in the absence of any commercial or financial relationships that could be construed as a potential conflict of interest.

## Publisher’s Note

All claims expressed in this article are solely those of the authors and do not necessarily represent those of their affiliated organizations, or those of the publisher, the editors and the reviewers. Any product that may be evaluated in this article, or claim that may be made by its manufacturer, is not guaranteed or endorsed by the publisher.

## Footnotes

1
https://abrc.osu.edu

2
http://www.ebi.ac.uk

3
http://www.premierbiosoft.com/primerdesign/

4
https://www.dna.affrc.go.jp/PLACE/?action=newplace
