# Supplementary material for: EjRAV1/2 Delay Flowering Through Transcriptional Repression of EjFTs and EjSOC1s in Loquat
Source: Front Plant Sci. 2021 Dec 31;12:816086. doi: 10.3389/fpls.2021.816086 (PMC8759039; doi:10.3389/fpls.2021.816086)
Supplement: Supplementary file 1 [file Data_Sheet_1.docx]

**
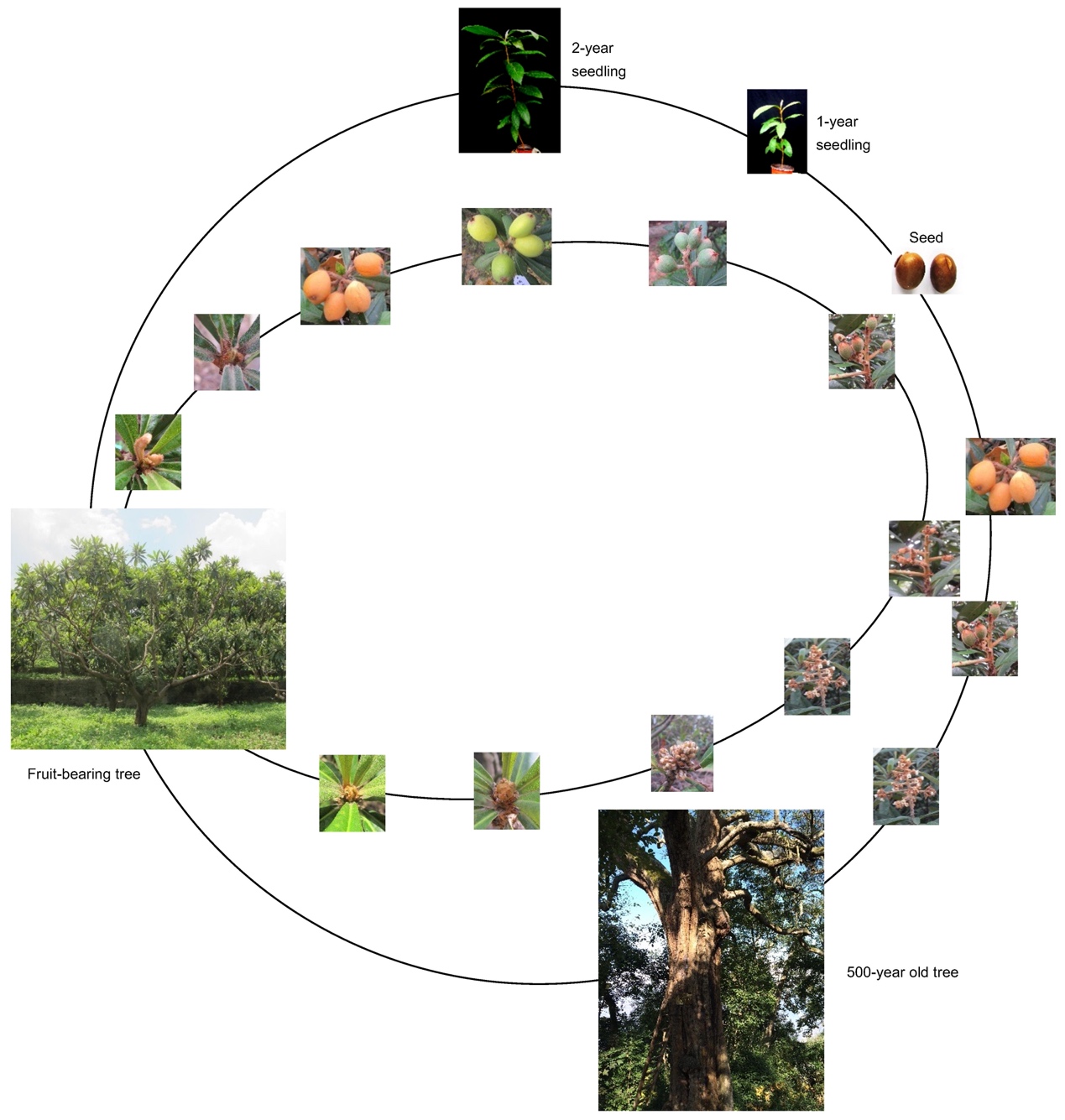
**

**Figure S1. The life history and annual growth cycle of cultivated loquat.** The outside circle shows the life history of cultivated loquat, from flowering, fruit set, fruit development, seed (reproductive phase), to seedling (juvenile phase), to fruit-bearing tree (adult phase), and finally to a 500-year-old flowering tree. The inside circle represents an annual growth cycle of cultivated loquat.

**
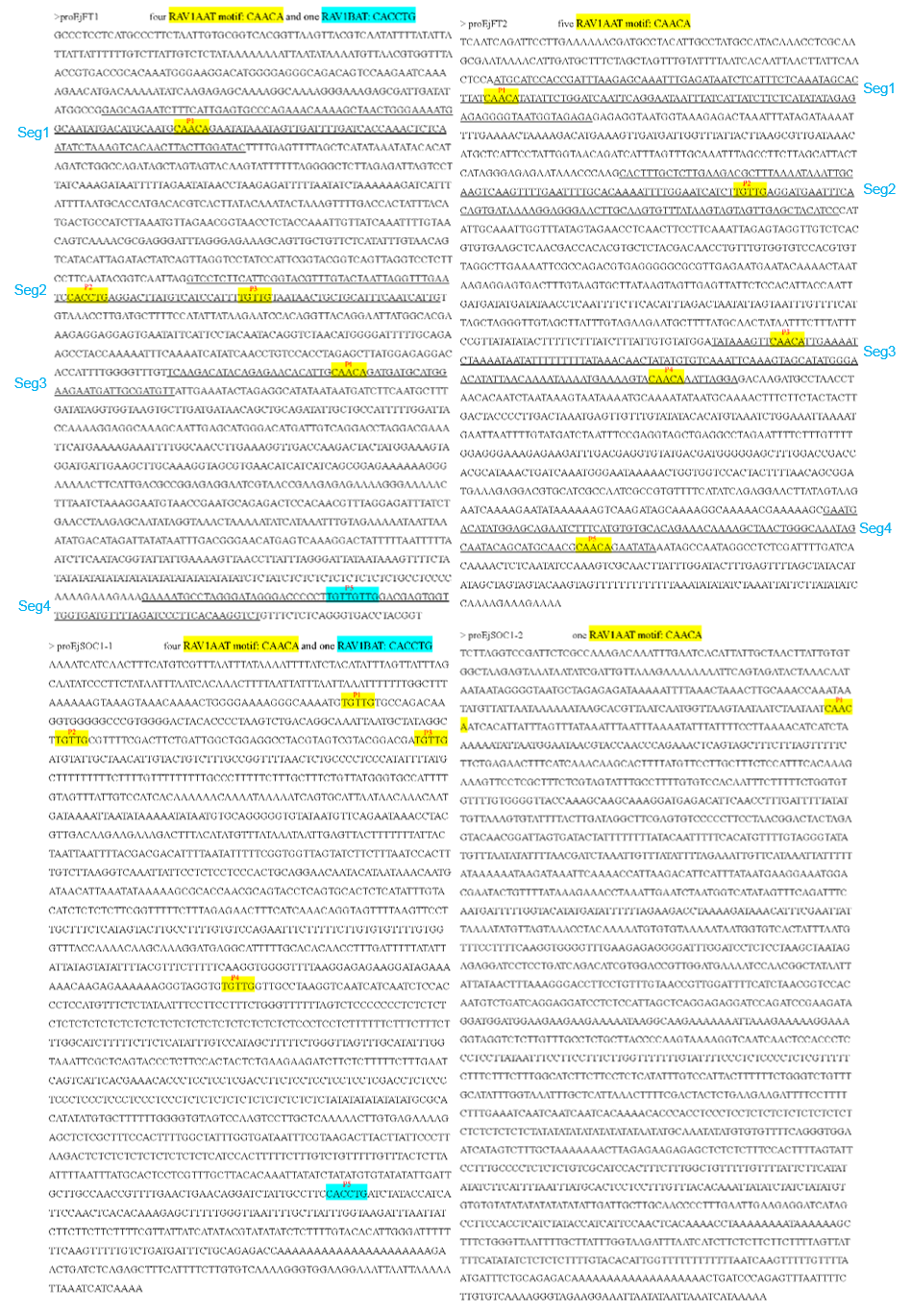
 Figure S2.** Promoter sequences of *EjFT1*, *EjFT2*, *EjSOC1-1* and *EjSOC1-2*. The RAV1AAT (CAACA) / RAV1BAT (CACCTG) motifs were highlighted in yellow or blue. The four underlined segments in both *proEjFT1* and *proEjFT2* were used for Y1H assays.

**
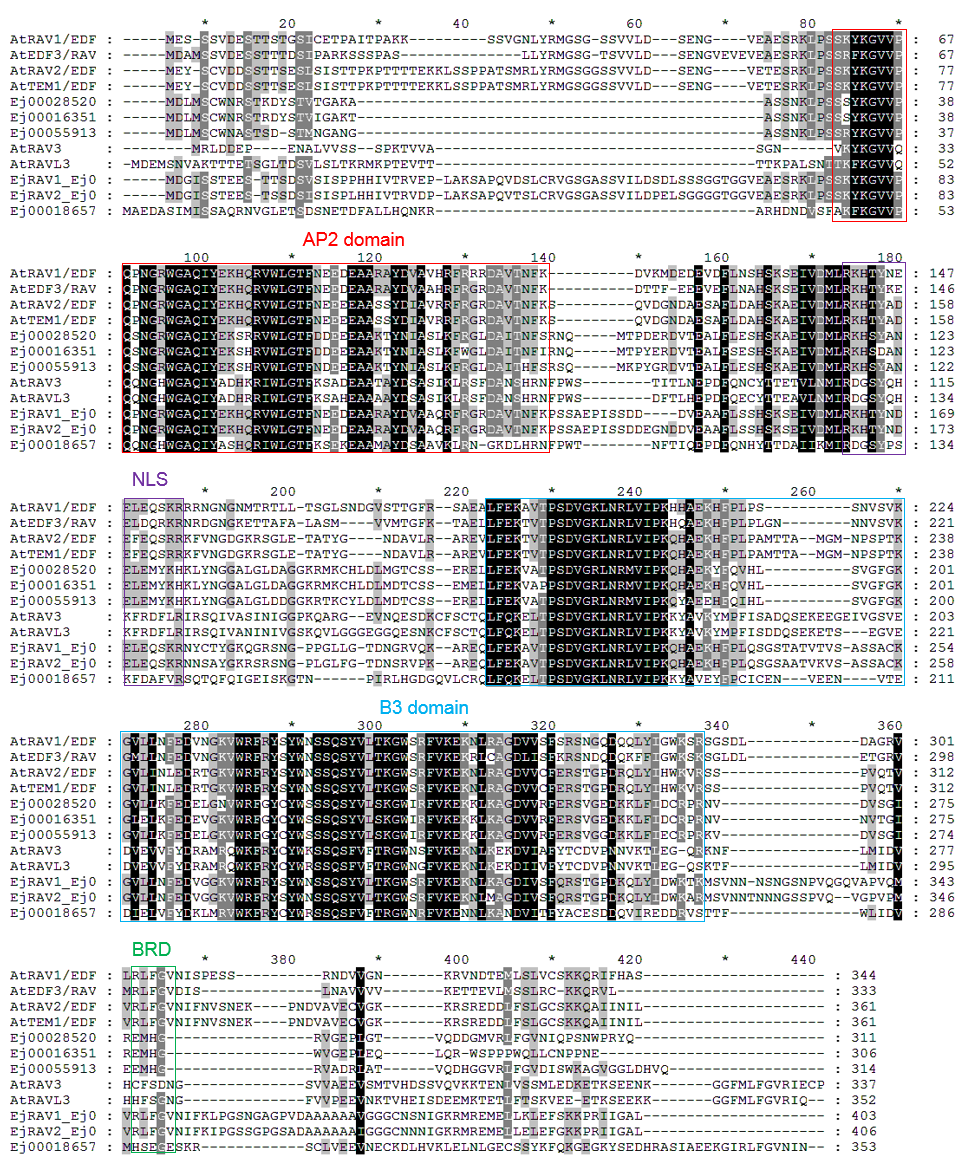
 Figure S3.** Sequence alignments of *EjRAV1* and *EjRAV2* with other *RAV* homologs. Amino acid sequences were aligned using Genedoc software. Both EjRAV1 and EjRAV2 proteins are characterized by an AP2 domain (in red box) and a B3 domain (in blue box); nuclear localization signal (NLS, in purple box) and B3 repression domain (BRD, in green box). Identical residues are shaped in black while conserved residues are in gray.


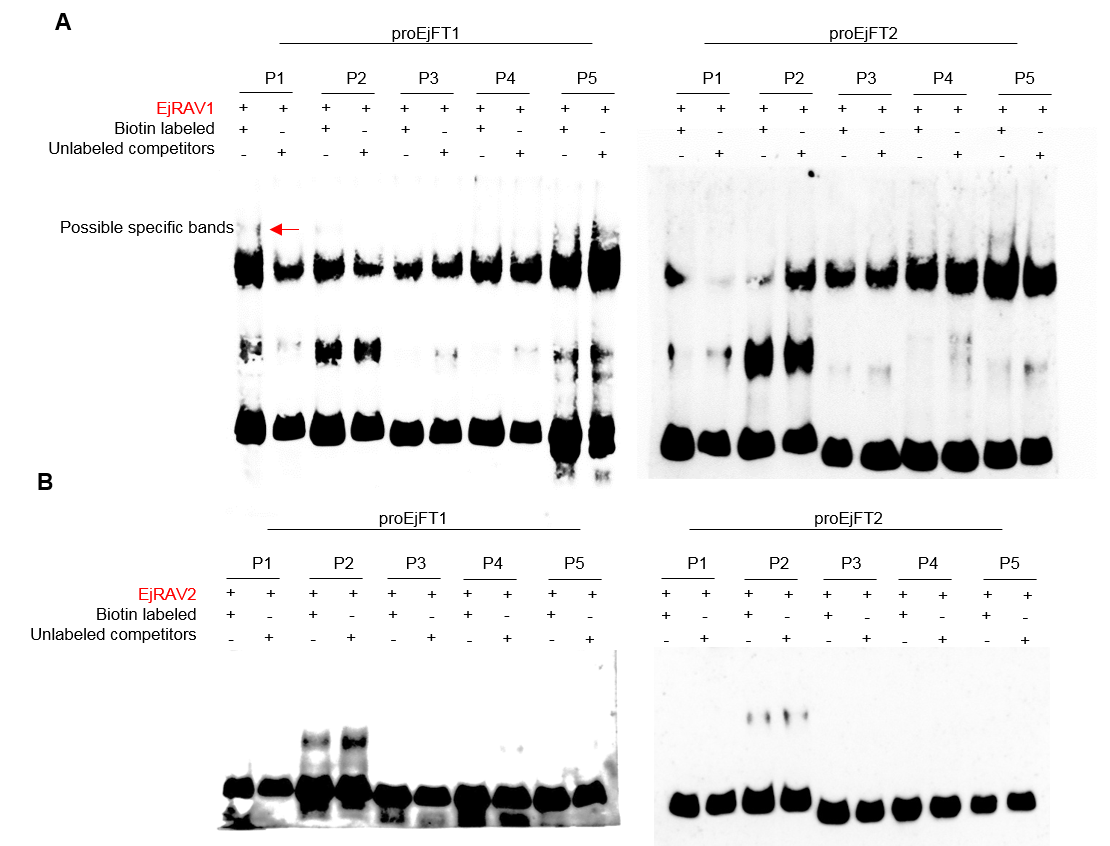


**Figure S4.** *In vitro* binding ability of EjRAV1/2 proteins to *EjFT* promoters by EMSA assays.


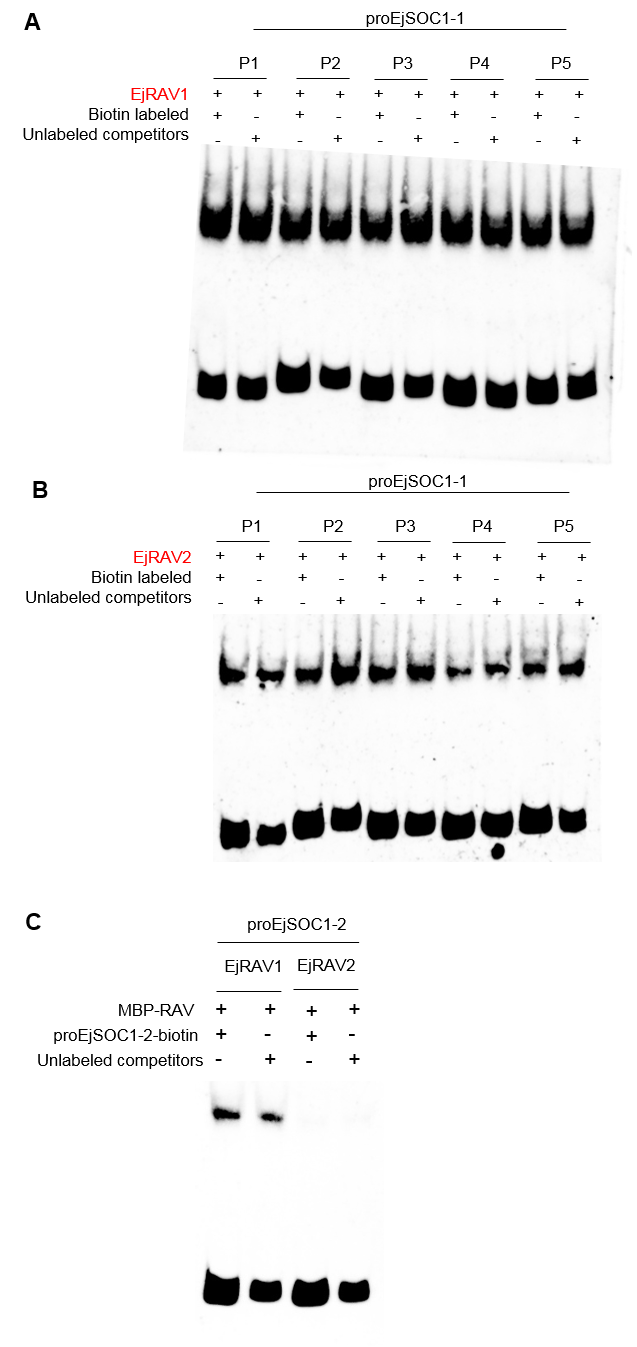


**Figure S5.** *In vitro* binding ability of EjRAV1/2 proteins to *EjSOC1* promoters by EMSA assays. No specific band was detected in all the EMSA assays.

**Table S1** Primers used in this study.

| Usages | Primer names | | Forward primer (5′→3′) | Reverse primer (5′→3′) |
| --- | --- | --- | --- | --- |
| Over-expression | 35S-EjRAV1 | | GTCGACGGTATCGATAAGCTTATGGATGGAATAAGCAGCACAG | TCCCCCGGGCTGCAGGAATTCCAAAGCTCCAATGATCCTTGGC |
|  | 35S-EjRAV2 | | GTCGACGGTATCGATAAGCTTATGGACGGAATAAGCAGCACAG | TCCCCCGGGCTGCAGGAATTCCAAAGCCCCAATGATCCTTGGC |
| Subcellular  localization | EjRAV1-GFP | | GTCGACGGTATCGATAAGCTTATGGATGGAATAAGCAGCACAG | TCCCCCGGGCTGCAGGAATTCCAAAGCTCCAATGATCCTTGGC |
|  | EjRAV2-GFP | | GTCGACGGTATCGATAAGCTTATGGACGGAATAAGCAGCACAG | TCCCCCGGGCTGCAGGAATTCCAAAGCCCCAATGATCCTTGGC |
| Q-RT-PCR | qEjRAV1 | | TGAGCTCCCACTCCAAGTCTG | CGACCGTTGTCCGTCCC |
|  | qEjRAV2 | | AGATTTACGAGAAGCACCA | GTCGTCGTTTCCTTCGTC |
|  | qEjAP1 | | TGATGCTCAAGTTGCTGTGG | TGCATGAATCTGTGGCGTAC |
|  | qEjFT1 | | ATTCCAGCAACAACCG | TGGCGAAACAGCACAAAA |
|  | qEjFT2 | | GGTTACCGATATTCCAGC | GCGAAACACCACCAAAA |
|  | qEjSCO1-1 | | GCGTTATCAGAAGCATGCGAAAGA | GCTCGGACGTTGTAGACGCTC |
|  | qEjSOC1-2 | | GAGCGATATCAGAATCACGC | GGATCGGACGTTGTTCACGC |
|  | qEjRPL18 | | ATGGGATTTGGCTTCGTTATC | AGAGTT TTGCTGGGATGGTG |
|  | qAtFT | | ACCCTGGTGCATACACTGTT | GGTGGAGAAGACCTCAGGAA |
|  | qAtSOC1 | | GTGATCTCCACTCAACAAAAA | CAACAAGAGAGAAGCAGCTTTA |
|  | qAtAP1 | | CATGGGTGGTCTGTATCAAGAAGAT | CATGCGGCGAAGCAGCCAAGGTT |
|  | qAtLFY | | ACGCCGTCATTTGCTACTCT | CTTTCTCCGTCTCTGCTGCT |
|  | qAtUBQ10 | | GGACCAGCAGCGTCTCATCTTCGCT | CTTATTCATCAGGGATTATACAAG |
| LUC/REN  assays | pSAK277- EjRAV1 | | ACTAGTGGATCCAAAGAATTCATGGATGGAATAAGCAGCACAG | GACTCTAGAAGTACTCTCGAGCTACAAAGCTCCAATGATCCTTG |
|  | pSAK277- EjRAV2 | | ACTAGTGGATCCAAAGAATTCATGGACGGAATAAGCAGCACAG | GACTCTAGAAGTACTCTCGAGCTACAAAGCCCCAATGATCCTTG |
|  | proEjFT1-0800 | | GTCGACGGTATCGATAAGCTTGCCCTCCTCATGCCCTTCTA | CGCTCTAGAACTAGTGGATCCACCGTAGGTCACCCTGAGAG |
|  | proEjFT2-0800 | | GTCGACGGTATCGATAAGCTTTCAATCAGATTCCTTGAAAAAAC | CGCTCTAGAACTAGTGGATCCTTTTCTTTCTTTTGGATATATAAG |
| EMSA | EjFT1-P1 | | TGGCAATATGACATGCAATGCAACAGAATATAAATAGTTGATTTTG | CAAAATCAACTATTTATATTCTGTTGCATTGCATGTCATATTGCCA |
|  | EjFT1-P1-mutant | | TGGCAATATGACATGCAATGTTTTTGAATATAAATAGTTGATTTTG | CAAAATCAACTATTTATATTCAAAAACATTGCATGTCATATTGCCA |
|  | EjFT1-P2 | | GTACTAATTAGGTTTGAATCCACCTGAGGACTTATGTCATCCATT | AATGGATGACATAAGTCCTCAGGTGGATTCAAACCTAATTAGTAC |
|  | EjFT1-P3 | | AGGACTTATGTCATCCATTTTGTTGTAATAACTGCTGCATTTC | GAAATGCAGCAGTTATTACAACAAAATGGATGACATAAGTCCT |
|  | EjFT1-P4 | | GACATACAGAGAACACATTGCAACAGATGATGCATGGAAGAATGA | TCATTCTTCCATGCATCATCTGTTGCAATGTGTTCTCTGTATGTC |
|  | EjFT1-P5 | | TAGGGATAGGGACCCCCTTGTTGTTGGACGAGTGGTTGGTGATGTT | AACATCACCAACCACTCGTCCAACAACAAGGGGGTCCCTATCCCTA |
|  | EjFT2-P1 | | CATTTCTCAAATAGCACTTATCAACATATATTCTGGATCAATTCAGG | CCTGAATTGATCCAGAATATATGTTGATAAGTGCTATTTGAGAAATG |
|  | EjFT2-P2 | | GCACAAAATTTTGGAATCATCTTGTTGAGGATGAATTTCACAGTGAT | ATCACTGTGAAATTCATCCTCAACAAGATGATTCCAAAATTTTGTGC |
|  | EjFT2-P3 | | GTGTATGGATATAAAGTTCAACATTGAAAATCTAAAATAAT | ATTATTTTAGATTTTCAATGTTGAACTTTATATCCATACAC |
|  | EjFT2-P4 | | CAAAATAAAATGAAAAGTACAACAAATTAGGAGACAAGATGCCT | AGGCATCTTGTCTCCTAATTTGTTGTACTTTTCATTTTATTTTG |
|  | EjFT2-P5 | | AGCAATACAGCATGCAACGCAACAGAATATAAATAGCCAATAGG | CCTATTGGCTATTTATATTCTGTTGCGTTGCATGCTGTATTGCT |
|  | EjSOC1-1-P1 | | GGAAAAGGGCAAAATGTGTTGTGCCAGACAAGGTGG | CCACCTTGTCTGGCACAACACATTTTGCCCTTTTCC |
|  | EjSOC1-1-P2 | | AATTAATGCTATAGGCTTGTTGCGTTTTCGACTTCTGATTGG | CCAATCAGAAGTCGAAAACGCAACAAGCCTATAGCATTAATT |
|  | EjSOC1-1-P3 | | GTAGTCGTACGGACGATGTTGATGTATTGCTAACATTGT | ACAATGTTAGCAATACATCAACATCGTCCGTACGACTAC |
|  | EjSOC1-1-P4 | | AAAGGGTAGGTGTGTTGGTTGCCTAAGGTCAATCATC | GATGATTGACCTTAGGCAACCAACACACCTACCCTTT |
|  | EjSOC1-1-P5 | | CAGGATCTATTGCCTTCCACCTGATCTATACCATCATTCCA | TGGAATGATGGTATAGATCAGGTGGAAGGCAATAGATCCTG |
|  | EjSOC1-2-P1 | | GGTTAAGTAATAATCTAATAATCAACAATCACATTATTTAGTT | AACTAAATAATGTGATTGTTGATTATTAGATTATTACTTAACC |
| Y1H assays | pGADT7 | EjRAV1 | GCCATGGAGGCCAGTGAATTCATGGATGGAATAAGCAGCACAG | CAGCTCGAGCTCGATGGATCCCAAAGCTCCAATGATCCTTGGC |
|  |  | EjRAV2 | GCCATGGAGGCCAGTGAATTCATGGACGGAATAAGCAGCACAG | CAGCTCGAGCTCGATGGATCCCAAAGCCCCAATGATCCTTGGC |
|  | pAbAi | FT1-seq1 | GAAAAGCTTGAATTC GAGCTC  GAGCAGAATCTTTCATTGAG | ATACAGAGCACATGCCTCGAGGTATCCAAGTAAGTTGTGAC |
|  |  | FT1-seq2 | GAAAAGCTTGAATTC GAGCTC  GTCCTCTTCATTCGGTACG | ATACAGAGCACATGCCTCGAGCAATGATTGAAATGCAGCAG |
|  |  | FT1-seq3 | GAAAAGCTTGAATTC GAGCTC  TCAAGACATACAGAGAACAC | ATACAGAGCACATGCCTCGAGAACATCGCAATCATTCTTCC |
|  |  | FT1-seq4 | GAAAAGCTTGAATTC GAGCTC  GAAAATGCCTAGGGATAGG | ATACAGAGCACATGCCTCGAGAGACCTTGTGAAGGGATC |
|  |  | FT2-seq1 | GAAAAGCTTGAATTC GAGCTC  ATGCATCCACCGATTTAAG | ATACAGAGCACATGCCTCGAGTCTCTACCATTACCCCTC |
|  |  | FT2-seq2 | GAAAAGCTTGAATTC GAGCTC  CACTTTGCTCTTGAAGACG | ATACAGAGCACATGCCTCGAGGGATGTAGCTCAACTACTAC |
|  |  | FT2-seq3 | GAAAAGCTTGAATTCGAGCTCTATAAAGTTCAACATTGAA | ATACAGAGCACATGCCTCGAGTCCTAATTTGTTGTACTT |
|  |  | FT2-seq4 | GAAAAGCTTGAATTCGAGCTC GAATGACATATGGAGCAG | ATACAGAGCACATGCCTCGAGTATATTCTGTTGCGTTGCA |

**Table S2.** Positions of RAV protein binding sites upstream of four flowering integrator gene promoters. The yellow shading shows CAACA motif and blue shading shows CACCTG motif, ‘-’showed no binding motifs.

| **proEjFT1** | **proEjFT2** | **proEjSOC1-1** | **proEjSOC1-2** |
| --- | --- | --- | --- |
| -1583~-1578 bp | -1616~-1612 bp | -1834~-1830 bp | -1919~-1915 bp |
| -1059~-1054 bp | -1281~-1217 bp | -1760~-1756 bp | _ |
| -1033~-1029 bp | -797~-793 bp | -1707~-1703 bp | _ |
| -782~-778 bp | -695~-691 bp | -965~-952 bp | _ |
| -73~-66 bp | -179~-175 bp | -272~-267 bp | _ |
